# Revisiting the Neighborhood: How L2 Proficiency and Neighborhood Manipulation Affect Bilingual Processing

**DOI:** 10.3389/fpsyg.2018.01860

**Published:** 2018-10-04

**Authors:** Kimberley Mulder, Walter J. B. van Heuven, Ton Dijkstra

**Affiliations:** ^1^Centre for Language Studies, for Language Studies, Radboud University, Nijmegen, Netherlands; ^2^School of Psychology, University of Nottingham, Nottingham, United Kingdom; ^3^Donders Institute for Brain, Cognition and Behaviour, Radboud University, Nijmegen, Netherlands

**Keywords:** bilingual word processing, hermit words, orthographic neighborhood size, lexical decision, progressive demasking

## Abstract

We conducted three neighborhood experiments with Dutch–English bilinguals to test effects of L2 proficiency and neighborhood characteristics within and between languages. In the past 20 years, the English (L2) proficiency of this population has considerably increased. To consider the impact of this development on neighborhood effects, we conducted a strict replication of the English lexical decision (ELD) task by van Heuven et al. (1998, Experiment 4). In line with our prediction, English characteristics (neighborhood size, word and bigram frequency) dominated the word and non-word responses, while the non-words also revealed an interaction of English and Dutch neighborhood size. The prominence of English was tested again in two experiments introducing a stronger neighborhood manipulation. In ELD and progressive demasking, English items with no orthographic neighbors at all were contrasted with items having neighbors in English or Dutch (‘hermits’) only, or in both languages. In both tasks, target processing was affected strongly by the presence of English neighbors, but only weakly by Dutch neighbors. Effects are interpreted in terms of two underlying processing mechanisms: language-specific global lexical activation and lexical competition.

## Introduction

A frequently used metaphor in monolingual and bilingual research is that of *lexical activation*. Upon the presentation of an input letter string, word candidates in the mental lexicon are assumed to become active depending on their overlap with the input and their frequency of usage. Most researchers nowadays hold that in bilinguals, word retrieval is initially determined by the formal overlap in letters rather than the language to which the word belongs. According to this ‘language non-selective lexical access’ view, in bilingual word reading, word candidates from both languages that are similar to the input are activated in parallel (for an overview of studies, see, e.g., Dijkstra, 2007; Dijkstra et al., 2010).

Most similar within and across languages are words that differ only in a single letter position. These are called ‘orthographic neighbors’ (Coltheart et al., 1977). Words can be neighbors within one language (e.g., *light* and *night* in English) or across languages (e.g., *night* in English and *nicht*, meaning ‘niece,’ in Dutch). Following a language non-selective access account, upon reading a word, orthographic neighbors from both target and non-target language are activated and influence target word processing. For example, reading the English word *wood* will activate, besides English form-similar words like *good* or *word*, Dutch neighbors like *rood* (meaning ‘red’) and *wond* (meaning ‘wound’). Thus, co-activation of lexical representations from different languages occurs if these share enough formal characteristics with the input letter string.

Furthermore, words of a higher frequency may become active more quickly than words of a lower frequency (in terms of an activation metaphor, the former would have a negative ‘resting level activation’ closer to zero, allowing them to become active more quickly). Because native language (L1) words are assumed to have been used more frequently than words from a second language (L2), on average this also holds for L1 vs. L2 words. As a consequence, L1 words are more competitive when they are activated than L2 words, providing an explanation for asymmetric effects in the word retrieval of unbalanced bilinguals, i.e., L1 exerting stronger effects on L2 than vice versa. The same reasoning can be applied to more proficient vs. less proficient bilinguals: The subjective frequency of L2 word usage is higher in the first group, resulting in stronger L2 effects.

Given that each word in a neighborhood (set of neighbors) has its own subjective frequency and associated representation strength, the summed activation of all neighbors in a particular language reflects the strength of the language the words belong to. This makes the manipulation of neighborhoods within and across languages well suited for assessing the relative strength of two languages of the bilingual.

The present neighborhood study had several aims. First, in Experiment 1 we investigated to what extent L2 proficiency differences can affect the occurrence of within- and between-neighborhood effects in L1 and L2. By fully replicating an earlier experiment by van Heuven et al. (1998), we tested the hypothesis that an increase in English (L2) proficiency in Dutch-English bilinguals shifts the relative contribution of their two languages toward English in English lexical decision (ELD) (see Introduction Experiment 1).

Second, in Experiment 2 we tested the effect of stimulus properties on neighborhood effects in a very similar ELD task by means of a stronger type of neighborhood size manipulation. Specifically, we introduced a manipulation in terms of *hermit words* that have no neighbors at all in one of the languages (see Introduction Experiment 2).

Third, in Experiment 3 we tested if the effects observed in ELD (Experiment 2) could be generalized across tasks by including the same materials in English progressive demasking (EPDM). This would also suggest that in our relatively English-proficient bilinguals, the native language Dutch (L1) does not exert strong effects in tasks in which only English (L2) words occur (see Introduction Experiment 3).

Fourth, by contrasting neighborhood effects for target items with few (Experiment 1) and with no (Experiments 2 and 3) neighbors, we wished to clarify both theoretical and empirical issues with respect to neighborhood studies. From a theoretical perspective, the comparison across item and task types allows us to analyze the processing mechanisms underlying performance in more detail. From an empirical perspective, such an analysis will help to clarify the puzzling finding of some fragile neighborhood effects in studies such as van Heuven et al. (1998) and Dirix et al. (2016).

To set the stage for a more detailed description of our experiments later, we will summarize the limited set of available bilingual neighborhood studies here. Studies on bilingual neighbors have been scarce and, as far as we know, restricted to van Heuven et al. (1998), Midgley et al. (2008), Grossi et al. (2012), Van Kesteren et al. (2012), Oganian et al. (2015), Dirix et al. (2016), and Oganian et al. (2016).

van Heuven et al. (1998) presented the first large study that examined effects of within- and between-language neighborhood size on bilingual word recognition by manipulating the number of English (L2) and Dutch (L1) orthographic neighbors in progressive demasking, generalized lexical decision, and language-specific lexical decision tasks as performed by Dutch–English bilinguals. When English target words had more orthographic neighbors in Dutch, this systematically resulted in slower response times, while a larger number of English neighbors produced facilitatory effects for English target words in progressive demasking and Dutch–English generalized lexical decision (in which a positive response is required for both Dutch and English words). Remarkably, this was not the case for language-specific ELD, for which a puzzling significant English neighborhood size effect of only 3 ms was reported (in the participant analysis only).

In fact, across the study as a whole, the observed effects were relatively large for the non-target language, which was the native language Dutch (see **Tables 2**, **3**, and **8** below). The between-language (i.e., Dutch) neighborhood size effects disappeared in monolingual English speakers processing the same materials, but for them facilitation arose for within-language (English) neighborhood size.

More recently, Midgley et al. (2008) provided electrophysiological evidence for cross-linguistic neighborhood effects. They specifically focussed on the N400, an EEG-component that is sensitive to semantic aspects of word processing (e.g., Kutas and Hillyard, 1980). N400 amplitude is assumed to reflect how easily a word can be semantically integrated into the context, be it a single word, a sentence, or a discourse (Kutas and Federmeier, 2000, p. 464). In addition, the amplitude of the N400 is found to be larger when target words have more semantic associates (Kounios and Holcomb, 1992). In a monolingual study on neighborhood effects, Holcomb et al. (2002). observed that words with a larger number of orthographic neighbors resulted in greater semantic activation and, as a consequence generate larger N400s (cf. Mulder et al., 2013). Following Holcomb and Grainger (2007), who argued that the N400 reflects the mapping of whole-word form representation onto semantics, Midgley et al. (2008) hypothesized that “larger N400s for words with many orthographic neighbors would reflect inhibition across activated lexical representations that leads to increased difficulty in settling on a unique form-meaning association.” This mechanism was hypothesized to hold for both within-language and between-language neighborhood effects.

ERP-recordings of highly proficient French-English bilinguals reading in French or English revealed that words with many between-language neighbors generated a more negative-going ERP waveform in the N400 region than words with few between-language neighbors. Moreover, the between-language neighborhood size effects in the N400 ERP-component arose earlier and were more widely distributed for L2 (English) target words than L1 (French) target words. The authors concluded that “words with more cross-language neighbors suffer from the co-activation of the lexical representations of these neighbors, as reflected in the typically longer RTs found to these stimuli in behavioral studies […]”.

Grossi et al. (2012) partially replicated these results in an ERP-study with English-Welsh bilinguals who performed a semantic categorization task on English and Welsh words. In late bilinguals, words with many between-language neighbors elicited more negative ERP amplitudes than words with few of them between 175 and 500 ms after word onset. In the 300–500 ms window, this effect interacted with language (English or Welsh). Early bilinguals showed a more complex pattern of early effects and no N400 effects. To explain their findings, the authors suggest that activation of between-language orthographic neighbors is sensitive to how bilinguals learn and use their languages.

Van Kesteren et al. (2012) studied Norwegian–English bilinguals in a mixed ELD task and a mixed Norwegian lexical decision task using English and Norwegian word stimuli that included language-specific letters (“smør,” “hawk”) and bigrams (“dusj,” “veal”). The number of neighbors in English and Norwegian in these tasks was systematically manipulated. This manipulation led to null-results of neighborhood size, possibly because other sub-lexical markers of language membership (i.e., language-specific letters and bigrams) were more prominently used by the bilinguals under consideration.

Dirix et al. (2016) conducted a generalized Dutch-English lexical decision experiment as well as a large-scale eye-tracking study in which Dutch–English participants read the Dutch (L1) or English (L2) version of a novel by Agatha Christie. The generalized lexical decision experiment was comparable in stimulus materials and several other respects (but not analysis) to van Heuven et al. (1998) (Experiment 3). In line with van Heuven et al. (1998) a mixed-effect model analysis yielded an inhibitory effect of Dutch neighborhood density on English RTs for words with low bigram frequency, and a higher error rate on English words with more cross-linguistic neighbors. Unexpectedly, this finding was not paralleled by a main effect of Dutch neighborhood density in Dutch lexical decision RTs, nor by any significant effect of English. The authors ascribed this discrepancy for Dutch (L1) words relative to the monolingual literature as due to the use of a generalized lexical decision task, “which creates a bilingual context different from a normal unilingual lexical decision task.” Although this suggests there may be interactions between the effects of L1 and L2 neighbors under particular task conditions, these were not further considered. Remarkably, neither van Heuven et al. (1998) in ELD, nor Dirix et al. in generalized Dutch-English lexical decision reported a straightforward English (L2) neighborhood effect on the RTs. The results of the study became even more puzzling in Experiment 2, because in natural English reading in a one-language context, the presence of between-language neighborhood effects was confirmed, but the effects were largely facilitatory (rather than inhibitory) in nature.

Finally, Oganian et al. (2015, 2016) observed between-language neighborhood size effects in naming and language decision of language-specific and language-ambiguous pseudo-words. They found that neutral pseudo-words were preferentially categorized to the language that was predominant in their orthographic neighborhood. In addition, they observed that the processing of L1-marked pseudowords but not L2-marked pseudowords were affected by the number of orthographic neighbors from the two languages. This suggests that perception of L2 markers was sufficient to trigger language decisions, whereas the activation of lexical neighbors seemed to influence the decision process for L1 marked pseudowords. Thus, the authors suggest that between-language activation of the L1 may be restricted to cases of sublexical ambiguity, whereas activation of lexical representations may concern especially the presented L2 when the associated orthographic patterns are illegal in L1.

In sum, with one exception, factorial studies on between-language neighborhood size indicate that bilingual word recognition in a non-native language is indeed sensitive to the numbers of words (neighbors) similar to the target word in both their languages. This validates the manipulation of neighborhood density as a marker of the relative contribution of two languages to bilingual word recognition. At the same time, the puzzling results for within- and between-language effects of L1 neighbors, and the potential sensitivity of effects to task demands (e.g., generalized lexical decision vs. language-specific lexical decision), call for further research. In the present paper, we first replicate the ELD task (Experiment 4) by van Heuven et al. (1998) with the present generation of the same bilingual participant population. Next, we report on an ELD task (Experiment 2) and a progressive demasking task (Experiment 3) with a stronger neighborhood manipulation in terms of *hermits*.

## Experiment 1: English Lexical Decision With Neighbors

To the best of our knowledge, no published study has yet replicated van Heuven’s et al. (1998) findings of bilingual neighborhood effects in ELD. We conducted an exact replication of the experiment by van Heuven et al. (1998, Experiment 4) 20 years later. This experiment involved a contrast between large and small neighborhoods for target items in both L1 and L2. In our study, a new generation of the Radboud University psychology student population was tested in Nijmegen. Because we had full access to the study of 1998, we were able to replicate the original experiment in the greatest detail, using exactly the same procedure and even identical stimulus lists.

In principle, two different predictions can be formulated with respect to the outcome of the replication. First, one might expect exactly the same result pattern as 20 years ago. However, models such as BIA/BIA+ propose that the activation of words depends on their frequency of usage. When participants possess a stronger proficiency in English (L2), the relative subjective frequency distribution of English and Dutch words shifts. Subsequent lexical activation differences might result in faster response times in an English task, and more prominent English and less prominent Dutch effects of neighborhood, bigram and word frequency. We are in favor of this second account, because there is abundant evidence to suggest that current Dutch students are more proficient in their L2 English than those 20 years ago. In 2001, a large survey by the European Commission, entitled “Europeans and their languages” (Special Eurobarometer 147, 2001, p. 16), reported that 52.1% of the Dutch claimed to have a ‘good’ level of English and 20.1% claimed a ‘very good’ level. The same question in Special Eurobarometer 386 (2012, p. T67) elicited claims of 58% ‘good’ and 32% ‘very good.’ According to this last survey (2012, p. 171), young people in Europe also judge themselves better on all dimensions of multilingual communication in a second language than older people (e.g., 41% vs. 20% of the two groups indicate they can follow English news reports via radio and television). In sum, there is a strong cross-generational difference in L2 proficiency.

In sum, we predict that in our present generation of Dutch psychology students, the relative strength of English to Dutch has increased (even when they can still be considered as late and unbalanced bilinguals). This should result in relatively strong effects of English in our Experiment 1, a replication of van Heuven et al. (1998), but also, even more clearly, in our later hermit experiments (including a stronger manipulation of neighborhood size).

### Method

#### Participants

Thirty-two Dutch L2 speakers of English (mean age 23.7 years old, *SD* = 3.34), mostly undergraduates at the University of Nijmegen, were paid or received course credits to take part in this experiment. All were highly proficient in English, having learned English from the age of 11 onward. All had normal or corrected-to-normal eyesight. Care was taken to select participants with the same characteristics as those in van Heuven et al. (1998).

#### Materials and Procedure

All stimulus materials were identical to those in van Heuven et al. (1998, Experiment 4). Stimulus characteristics are summarized in **Table 1**. In van Heuven et al. (1998) the numbers of neighbors in each language were calculated following Coltheart et al. (1977). The item set consisted of 20 word and 40 non-word items in each condition. Stimulus lists, including stimulus order, in the present experiment were identical to those in the earlier study. The procedure followed was also identical, except that as a background survey the present experiment involved the Lextale task (Lemhöfer and Broersma, 2012) to assess English proficiency, and two language background questionnaires (one of which was identical to that used in van Heuven et al., 1998).

**Table 1:** Stimulus characteristics in the neighbor manipulation (van Heuven et al., 1998, Experiment 4; Experiment 1) and the hermit manipulation (Experiments 2 and 3).

	Stimulus category for Neighbor and Hermit manipulation	English N Neighbors	Dutch N Neighbors	English N Hermits	Dutch N Hermits
Word					
	Complete hermit/Small E-Small D	1.2	1.0	0	0
	Only Dutch neighbors/Small E-Large D	1.15	3.5	0	2.5
	Only English neighbors/Large E-Small D	3.5	0.95	4.2	0
	Neighbors in English and Dutch/Large E-Large D	3.6	3.5	5.1	3.4
Non-word					
	Complete hermit/Small E-Small D	1.0	1.0	0	0
	Only Dutch neighbors/Small E-Large D	1.0	3.5	0	3.6
	Only English neighbors/Large E-Small D	3.5	1.0	3.9	0
	Both English and Dutch/Large E-Large D	3.5	3.5	4.8	3.7


Participants performed an English visual lexical decision task, which was programmed in *Psychopy* and run on an HP Compaq Intel Core 2 computer with LCD monitor and a refresh rate of 120 Hz. The experimental set-up and stimulus presentation (font size and type of stimuli, background color, instructions, trial structure, etc.) were identical to those in van Heuven et al. (1998).

### Results

The mean participant and item accuracy was 92.37, and 93.81%, respectively. One participant (47.8% correct) and one item (61.29% correct) that had an error rate above 30% were removed (e.g., *keen*). Finally, errors and RTs outside the range of 2.5 SD from the item and participant mean were removed. **Tables 2**, **3** present the mean RTs, standard deviations, error rates, and neighborhood effects for different word and non-word types. For a comparison of neighborhood effects based on different neighborhood size contrasts, the mean RTs of the lexical decision data of Experiment 4 of van Heuven et al. (1998) are also presented^1^.

**Table 2:** Mean RTs (in ms), standard deviations, error rates, and neighborhood effects for English word stimuli of English Lexical Decision in van Heuven et al. (1998, Experiment 4), our replication study (Experiment 1), and Experiment 2 with hermits.

Language effect in van Heuven et al. (1998): Words	Large English	Small English	Effect size for English	Total effect size for English
Large Dutch	585 (69, 12.1)	583 (74, 12.1)	2	
Small Dutch	561 (70, 4.8)	564 (73, 9.5)	-3	-0.5
Effect size for Dutch	24	19		
Total effect size for Dutch	21.5			

**Language effect for neighbors in Experiment 1: Words**	**Large English**	**Small English**	**Effect size for English**	**Total effect size for English**

Large Dutch	524 (100, 8.8)	527 (102, 7.2)	-3	
Small Dutch	523 (98, 6.2)	525 (100, 7.6)	-2	-2.5
Effect size for Dutch	1	2		
Total effect size for Dutch	1.5			

**Language effect for hermits in Experiment 2: Words**	**Large English**	**No English**	**Effect size for English**	**Total effect size for English**

Large Dutch	594 (61, 6.1)	635 (64, 12.8)	-41	
No Dutch	599 (66, 5.9)	615 (65, 9.6)	-16	-28.5
Effect size for Dutch	-5	20		
Total effect size for Dutch	7.5			


**Table 3:** Mean RTs (in ms), standard deviations, error rates, and neighborhood effects for English non-word stimuli of English Lexical Decision in van Heuven et al. (1998, Experiment 4) and our replication study (Experiment 1), and Experiment 2 with hermits.

Language effect in van Heuven et al. (1998): Non-words	Large English	Small English	Effect size for English	Total effect size for English
Large Dutch	651 (94, 9.5)	635 (94, 4.0)	16	
Small Dutch	642 (99, 8.1)	626 (93, 3.5)	16	16
Effect size for Dutch	9	9		
Total effect size for Dutch	9			

**Language effect for neighbors in Experiment 1: Non-words**	**Large English**	**Small English**	**Effect size for English**	**Total effect size for English**

Large Dutch	592 (104, 6.3)	567 (97, 2.8)	25	11
Small Dutch	577 (108, 4.1)	580 (106, 3.7)	-3	
Effect size for Dutch	15	-13		
Total effect size for Dutch	1			

**Language effect for hermits in Experiment 2: Non-words**	**Large English**	**No English**	**Effect size for English**	**Total effect size for English**

Large Dutch	685 (84, 10.2)	638 (76, 2.8)	47	
No Dutch	694 (84, 7.5)	652 (82, 4.0)	42	44.5
Effect size for Dutch	-9	-14		
Total effect size for Dutch	-11.5			


Inspection of the distribution of the response latencies revealed non-normality. A comparison of a log-transform and an inverse transform (-1000/RT) revealed that the inverse RT was most successful in reducing the non-normality. The word and non-word data were then analyzed with linear mixed effects models with subject and item as crossed random effects. Similar to van Heuven et al. (1998) the following factorial predictors were considered in the analyses: *English Neighbors* (Large or Small) and *Dutch Neighbors* (Large or Small). Further, we added the following continuous predictors to our model in a step-wise inclusion procedure: *English Frequency* (log-transformed subtitle frequency, SBTLWF; Brysbaert and New, 2009), *English Bigram Frequency* and *Dutch Bigram Frequency* (both log-transformed; Duyck et al., 2004), *Trial* (the rank of the item in the stimulus list), and *Previous RT* (the log-transformed response latency at the previous trial).

We included English and Dutch bigram frequency as factors in our analyses, because bilinguals can use sublexical statistical information such as bigram frequency to identify language membership (Oganian et al., 2016). In addition, in the study by Dirix et al. (2016), this variable contributed relatively strongly to the obtained data patterns. For the random effects structure, we considered random slopes by participant for all predictors mentioned above.

To obtain the best fitting model, we performed a stepwise variable selection procedure in which one predictor was added at a time. For each significant predictor or interaction, it was evaluated whether inclusion of this predictor or interaction resulted in a better model (i.e., had a lower AIC compared to when this predictor was not part of the model). Next, the final model was trimmed by removing any remaining extreme outliers (defined as data points with standardized residuals exceeding 2.5 standard deviation units).

**Tables 4**, **5** summarize the final models for the word and non-word analyses, respectively. The final regression model for the word data in **Table 4** revealed a significant interaction between *English Neighbors* and *English Bigram Frequency*, showing that response latencies are faster when the English bigram frequency and English neighborhood size is large compared to when the English neighborhood size is small. **Figure 1** displays this interaction. Furthermore, *English Frequency* had a facilitatory effect on RT. Finally, the effect of *PreviousRT* shows that responses become slower when the response to the previous item was also slow.

**Table 4:** Final model for the word data in Experiment 1 (English Lexical Decision with neighbors).

Fixed effects	Estimate	Standard error	*t*-value
Intercept	-2.83363	0.26097	-10.858
Previous RT	0.13605	0.02863	4.753
English Neighbors	0.38399	0.19681	1.951
English Bigram Frequency	0.02403	0.02231	1.077
English Frequency	-0.05862	0.01091	-5.371
English Neighbors by English Bigram Frequency	-0.04761	0.02379	-2.001

**Random effects**	**Variance**	**Standard deviation**	

Item (Intercept)	0.01356	0.1164	
Participant (Intercept)	0.02457	0.1567	
Residual	0.06380	0.2526	


**t* > 1.96 or <-1.96 is significant. Final model: invRT∼ Previous RT + English Neighbors ^∗^ English Bigram Frequency + English Frequency + (1| participant) + (1| item). On the intercept are words with a small number of English and Dutch neighbors.*

**Table 5:** Final model for the non-word data in Experiment 1 (English Lexical Decision with neighbors).

Fixed effects	Estimate	Standard error	*t*-value
Intercept	-2.76443	0.16752	-16.502
Previous RT	0.15861	0.02607	6.083
English Neighbors	-0.01016	0.02750	-0.370
Dutch Neighbors	-0.03350	0.02742	-1.222
English Neighbors: Dutch Neighbors	0.07942	0.03888	2.042

**Random effects**	**Variance**	**Standard deviation**	

Item (Intercept)	0.005621	0.07497	
Participant (Intercept)	0.017120	0.13084	
Residual	0.053643	0.23161	


**t* > 1.96 or <-1.96 is significant. Final model: invRT∼ Previous RT + English Neighbors ^∗^ Dutch Neighbors + (1| participant) + (1| item). On the intercept are words with a small number of English and Dutch neighbors.*

**FIGURE 1:**
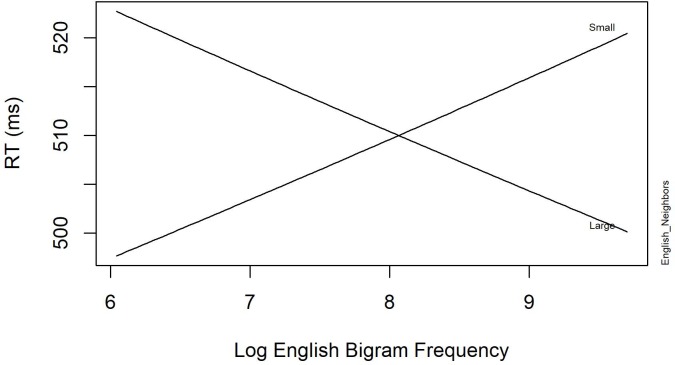
The significant interaction between English Neighbors and Log English Bigram Frequency in English Lexical Decision with neighbors (Experiment 1).

**FIGURE 2:**
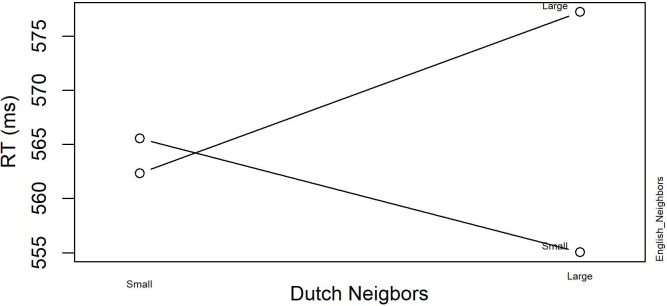
The significant interaction between English Neighbors and Dutch Neighbors in English Lexical Decision with neighbors (Experiment 1).

The final model for the non-word data revealed a significant interaction between *English Neighbors* and *Dutch Neighbors*, indicating that responses times are slower when both English and Dutch neighborhood size are large. **Figure 2** displays this interaction. The inhibitory effect of *Previous RT* shows that responses become slower when the response to the previous item was also slow.

Finally, as **Tables 2**, **3** reveal that our participants are considerably faster than the participants in van Heuven et al. (1998) effects of Dutch neighborhood size might occur only in the slower participants However, a median split of our data in a fast (mean RT = 519) and slow group (mean RT = 590) again revealed no effects of Dutch neighborhood density in both groups.

### Discussion

The results of our ELD experiment, a replication of van Heuven et al. (1998, Experiment 4), are in line with our prediction that Dutch-English bilinguals have become better in English in the last 20 year. With respect to English and Dutch neighborhood effects and (sub)lexical factors, a shift toward English was observed in the response patterns for words and non-words. Only a small interaction effect of English and Dutch neighbors was observed in the non-words. We conclude that, apart from stimulus properties, relative L2 proficiency is an important determinant of neighborhood effects, explaining in part why studies involving similar tasks and designs may still obtain different results^2^. Thus, future studies should be even more strict in their experimental manipulations and characterize their participant groups in as much detail as possible.

We consider the prominence of English in our participants to be a consequence of more intensive contact of the present generation of students with English in school, due to English university books, and/or due to the English-oriented Internet. Since 1993, Dutch children start to acquire English as their second language at least 2 years earlier than before, at the age of 10–11 rather than 12–13, due to a change in school systems and the strong increase in so-called ‘bilingual schools’ (Van Hell, 1998; Edelenbos and Vinjé, 2000). In addition, in the last decade, English has become default for Dutch students when they are searching the internet, and many Bachelor programs at Dutch universities are now taught in English. This has even instigated a debate on the role Dutch should play in the scientific education in various disciplines. We propose that this Dutch trend toward increased bilingualism, signaled by De Swaan, 2001 (p. 202), continues strongly today. Seen in this light, the comparison of the two studies shows how societal changes affect the L2 proficiency of participant populations, resulting in systematic differences in observed data patterns over time.

When the present generation of Dutch–English bilinguals have a stronger representation of English, its effect should become even more prominent when a stronger neighborhood manipulation is applied. Such a manipulation is that of hermit neighbors in Experiment 2 (English lexical decision) and Experiment 3 (English progressive demasking).

## Experiment 2: English Lexical Decision With Hermits

In Experiment 1, we manipulated neighborhood size in terms of many vs. few neighbors. All studies so far used this specific manipulation, although they differed in other respects (e.g., participant groups, language pairs, and experimental techniques). However, Bowers et al. (2005) have argued that the critical and optimal neighborhood contrast to consider is not between words with many and few neighbors, but between words with one or more neighbors and with no neighbors. They pointed out that word processing models like IA and SOLAR predict little difference between words with few and many neighbors (Davis and Andrews, 1996), because there is no additional competition for words with many neighbors due to a normalization of the total amount of activity at the word level. Thus, in order to have a pure measurement of neighborhood size effects, words with one or more neighbors should be contrasted with words with no neighbors, the so-called *hermit* words.

Bowers et al. (2005) addressed this issue by having monolingual English participants learn new words (e.g., *banara*) that were neighbors of familiar hermit words (e.g., *banana*) and respond to these familiar words in a semantic categorization task. They observed that repeated exposure to the novel neighbor word made it more difficult to semantically categorize the familiar words. Interference effects even became larger with more training on the novel words. The authors concluded that the impact of the new neighbors on semantically classifying the hermit words is likely to reflect lexical competition and is in accordance with the predictions made by the IA and SOLAR models.

To include the strongest test of neighborhood effects possible in our study, we therefore contrasted word conditions with many or no neighbors at all in English and Dutch in Experiments 2 and 3. This resulted in four conditions: English words without any orthographic neighbors in English or Dutch, referred to as *complete hermits*; English words with neighbors in Dutch but not in English; English words with only English and no Dutch neighbors; and English words with neighbors in both languages. By comparing complete hermits to words that are hermits only in Dutch, we can directly assess the role of Dutch neighbors, while a comparison to hermits only in English should directly reflect effects of English neighbors. This should allow us to test the occurrence of between-language neighborhood size effects with a more pure contrast of neighborhood size than before.

As tasks, we chose ELD and progressive demasking, because in van Heuven et al. (1998; Experiments 1 and 4), both of these tasks included exclusively English (L2) words, while the non-words in ELD were also derived from English. In this case, it can be clearly seen to what extent the native language of our participants, Dutch, is able to affect non-native English language processing. A further reason to opt for the language-specific lexical decision task was that, in contrast to predictions, van Heuven et al. (1998) observed only a small effect of English neighbors in their English target word responses [(significant only in the participant analyses, F1); see **Table 2**]. It is therefore important to demonstrate that within-language neighborhood effects of English *can* be obtained by contrasting words with many and no neighbors. Finally, by applying the same neighborhood contrast to non-word stimuli in lexical decision, we cannot only collect control data for comparison with the word data, but also obtain more insight into neighborhood effects for targets without lexical representation and linked to a no-response.

Furthermore, while language-specific lexical decision requires a forced choice between two responses, in a paradigm such as progressive demasking, a target word must be identified in a background of noise (see Keuleers and Brysbaert, 2012). Thus, by conducting a progressive demasking experiment involving the same stimulus materials, we can assess the effect of task differences on the obtained result patterns. This will also help to better understand which mechanisms underlie performance in different language and task situations. Finally, if Experiments 2 and 3 with the hermit manipulation both demonstrate that, relative to Dutch (L1) neighbors, English (L2) neighbors exert a stronger effect on the RTs than in the replication by van Heuven et al. (1998, Experiment 1), this provides evidence that neighborhood effects are sensitive to subtle properties of the stimulus materials, in particular the degree to which they activate the background language Dutch (the strong L1) in a task requiring responses to the target language English (the weaker L2).

Note that in our hermit experiments, we applied exactly the same method for calculating within-language and between-language neighbors as van Heuven et al. (1998) and also preserved their four experimental neighborhood conditions. The only difference was that in our ‘small’ English and ‘small’ Dutch neighborhood conditions, neighborhood density was zero instead of one or more. This allowed us to see whether a stronger neighborhood contrast would lead to the same pattern of effects. Furthermore, the hermit conditions allowed a purer assessment of the independent effects of English and Dutch neighborhood density, particularly in the comparison of words with no neighbors at all to words with neighbors in one language only.

### Method

#### Participants

Forty-one Dutch L2 speakers of English (mean age 22.6 years old, *SD* = 2.58), mostly undergraduates at the University of Nijmegen, were paid or received course credits to take part in this experiment. All were highly proficient in English, having learned English from the age of 11 onward. All had normal or corrected-to-normal eyesight.

#### Materials

For Experiments 2 and 3, we selected 105 English four- and five-letter words from the CELEX lexical database (Baayen et al., 1995). All words were mono-morphemic non-cognate words of 1–2 syllables with a frequency of at least 2 occurrences per million. The numbers of within-language (English) and between-language (Dutch) orthographic neighbors (based on neighboring word forms) were extracted from CELEX for English and Dutch (following van Heuven et al., 1998, by using Coltheart et al., 1977). Note that the CELEX neighborhood size count does not include deletion or addition neighbors. The number of English neighbors was checked with the OrthoN measure from the English Lexicon Project (Balota et al., 2007) for English. The values largely correspond to those of OrthoN, with the exception that certain acronyms (such as WOHD for “World Oral Health Day” as a neighbor for WOOD) or very low frequency words (which are likely not to be known by our participants) are not included in the CELEX count.

The selected word items were divided into four stimulus categories: 30 English words without neighbors in either English or Dutch (complete hermits, e.g., *abbey*), 15 English words without neighbors in English, but with neighbors in Dutch (e.g., *bias* with Dutch neighbors *baas*), 30 English words with neighbors in English but not in Dutch (e.g., *faint* with English neighbors *paint* and *saint*), and 30 English words with neighbors in both languages (e.g., *wood* with English neighbors such as *good* and *mood* and Dutch neighbors such as *woud* and *rood*). Unfortunately, the asymmetry in the number of items in the stimulus categories could not be resolved due to the limited number of existing English words that have neighbors in Dutch but not in English. The items of the four categories were matched on log-transformed values of *SUBTLWF* (English SUBTLEX word frequency per million; Brysbaert and New, 2009), English and Dutch bigram frequency, and length. We chose to match the items on the log-transformed values of SUBTLEX rather than on the CELEX frequency values, as the former have been shown to better predict response latencies (Brysbaert and New, 2009) and reflect a more up-to-date measure of frequency. Furthermore, the stimulus categories containing items with English or Dutch neighbors were matched on mean number of neighbors in these languages. Moreover, the stimulus categories containing items with English neighbors and the categories containing no English neighbors were matched on OLD-20 (i.e., the mean of the closest 20 Levensthein Distance orthographic neighbors, see Balota et al., 2007; Yarkoni et al., 2008).

The same contrast in within-language and between-language neighborhood size for the word items was applied to non-words. The four non-word categories each contained 30 items and were matched to each other and to the four word categories on length, English and Dutch bigram frequency, and number of English and Dutch neighbors. The Appendix lists all word and non-word items. **Table 1** contrasts the neighborhood characteristics of Experiment 1 (identical to van Heuven et al., 1998) and Experiments 2 and 3. **Table 1** shows that the number of neighbors in the large N conditions are fairly similar across studies. This allows for a clean comparison of within- and between-language effects in both studies. Additional item properties of our stimuli are presented in **Table A1** of the **Appendix**.

Finally, to obtain an equal number of words and non-words, we added 45 word fillers and 30 non-word fillers to the item set. These were matched on length, English and Dutch bigram frequency to the word items. This resulted in a total stimulus set of 300 items.

Items were presented in two blocks. Presentation order of items within a block was randomized individually with the restriction that no more than three words or non-words could follow each other directly.

#### Procedure

Participants performed an English visual lexical decision task, which was programmed in *Presentation* v13.0 (Neurobehavioral Systems)^3^ and run on an HP Compaq Intel Core 2 computer with a LCD monitor and a refresh rate of 120 Hz. Participants were seated at a table at a 60 cm distance from the computer screen. Stimuli were presented in black capital letters (24 points) in font Arial in the middle of the screen on a white background. Participants were tested individually in a sound-proof room. They first read an English instruction that asked them to push the ‘yes’ button if a presented letter string was an existing English word and the ‘no’ button if it was not. They were asked to react as accurately and quickly as possible.

Each trial started with the presentation of a black fixation point ‘+’ in the middle of the screen for 700 ms. After an empty black screen of 300 ms, the target stimulus was presented. It remained on screen until the participant responded or a maximum of 1500 ms passed by. The target stimulus disappeared when the participant pressed a response button, or after 1500 ms. Following an empty screen of 500 ms, a new trial was started.

The experiment was divided in two blocks of equal length. The first block was preceded by 20 practice trials. Next, the participant could ask questions before continuing with the test trials. The two blocks each contained 150 experimental trials. Each block began with three dummy trials to avoid lack of attention during the beginning of the two blocks. The end of the first block was indicated by a pause screen. The experiment took approximately 16 min.

After the experiment, participants performed the XLEX-task (Meara and Milton, 2003) to obtain a general measure of proficiency in terms of vocabulary knowledge. The mean score on the XLEX-task was 4275 (range 3000–5000). Moreover, participants were asked to fill out an off-line pencil-and-paper questionnaire about their level of proficiency in English. Participants reported to have been in regular contact with English from the age of 11 onward, and to read English books or texts on a regular basis. Based on their scores on the XLEX-task and their answers from the questionnaire, all participants were considered as highly or intermediately proficient.

### Results

Data cleaning was first carried out based on the error rate for participants. Mean participant accuracy on word items was 90% (range 66–99%) and 91% (range 44–99%) on non-word items. The data from three participants with an error rate of 25% or more on word or non-word items were removed from the data set. Next, items that elicited errors in more than 35% of the trials were removed from the data [mean item accuracy on the words 90% (range 29–100% and non-words 91%, range 49–100%)]. This resulted in the exclusion of three word items (*lunar, lapse*, and *gorge*) and four non-word items (*goast, hount, sooth, lawer*). Moreover, *groap*, *mair*, and *pleat* were removed because they are to some extent homophonic. After removal of these items, we were left with 8170 data points. RTs from incorrect responses or null responses were removed from the data (7.2% of the 8170 data points, of which 17.7 and 8.2% on complete hermits words and non-words, 11.3 and 5.8% for words and non-words with only Dutch neighbors, 10.8% and 14.5% on words and non-words with only English neighbors, and 11.5 and 20.2% for words and non-words with Dutch and English neighbors). Outlier RTs that were above or below 2.5 SD from the item or participant mean (4.3% of the remaining data points) were removed from the data. This resulted in a data set with 7354 data points. **Tables 2**, **3** present the mean RTs, standard deviations, error rates, and neighborhood effects for different word and non-word types. For a comparison of neighborhood effects based on different neighborhood size contrasts, the mean RTs of the lexical decision data of Experiment 4 of van Heuven et al. (1998) are also presented.

Inspection of the distribution of the response latencies revealed non-normality. A comparison of a log-transform and an inverse transform (-1000/RT) revealed that the inverse RT was most successful in reducing the non-normality. The word and non-word data were then analyzed with linear mixed effects models with subject and item as crossed random effects. The following factorial predictors were considered in the analyses: *English Neighbors* (yes or no) and *Dutch Neighbors* (yes or no). Further, we considered the same predictors as used in Experiment 1: *English Frequency* [log-transformed subtitle frequency (SBTLWF), Brysbaert and New, 2009], *English Bigram Frequency* and *Dutch Bigram Frequency* (both log- transformed; Duyck et al., 2004), *Trial* (the rank of the item in the stimulus list), and *Previous RT* (the inverse-transformed response latency at the previous trial).

**Tables 6**, **7** summarize the final models for the word and non-word analyses, respectively. **Figure 3** displays the main effect of *English Neighbors* and the significant interaction of *English Frequency* and *English Bigram Frequency* in the model on the word data.

**Table 6:** Final model for the word data in Experiment 2 (English Lexical Decision with hermits).

Fixed effects	Estimate	Standard error	*t*-value
Intercept	-0.624	0.533	-1.170
Previous RT	0.077	0.015	5.130
Trial	0.001	0.008	0.183
English Frequency	-0.379	0.151	-2.506
English Neighbors	-0.072	0.022	-3.331
Dutch Neighbors	0.001	0.021	0.062
English Bigram Frequency	-0.083	0.006	-1.365
English Frequency by English Bigram Frequency	0.035	0.017	2.013

**Random effects**	**Variance**	**Standard deviation**	

Item (Intercept)	0.009	0.094	
Participant (Intercept)	0.023	0.152	
Trial (Participant)	0.002	0.040	
Residual	0.059	0.243	


**t* > 1.96 or <-1.96 is significant. Final model: invRT∼ Previous RT + Trial + English Neighbors + Dutch Neighbors + English Frequency ^∗^ English Bigram Frequency + (1| participant) + (1| item). On the intercept are words with no English and Dutch neighbors.*

**Table 7:** Final model for the non-word data in Experiment 2 (English Lexical Decision with hermits).

Fixed effects	Estimate	Standard error	*t-value*
Intercept	-1.692	0.161	-10.538
Previous RT	0.118	0.015	8.164
Trial	-0.017	0.006	-2.722
English Neighbors	0.091	0.017	5.254
Dutch Neighbors	-0.026	0.017	-1.501
English Bigram Frequency	0.033	0.018	1.827

**Random effects**	**Variance**	**Standard deviation**	

Item (Intercept)	0.007	0.083	
Participant (Intercept)	0.026	0.162	
Trial (Participant)	0.001	0.030	
Residual	0.053	0.232	


**t* > 1.96 or <-1.96 is significant. Final model: invRT∼ Previous RT + Trial + English Neighbors + Dutch Neighbors + English Bigram Frequency + (1| participant) + (1| item). On the intercept are words with no English and Dutch neighbors.*

**FIGURE 3:**
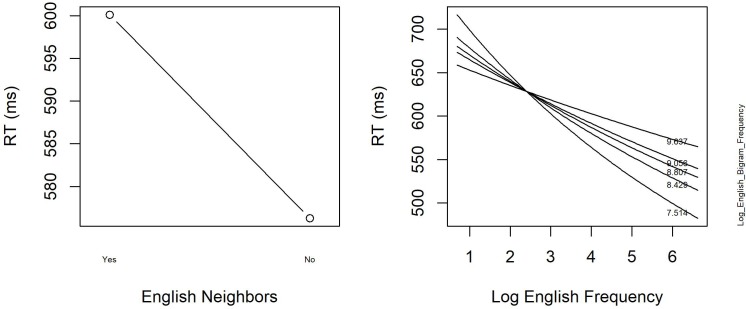
The significant main effect of English Neighbors and the interaction between Log English Frequency and Log English Bigram Frequency in English Lexical Decision with hermits (Experiment 2).

The final regression model for the word data in **Table 6** revealed a facilitatory main effect of *English Neighbors* in our study. Responses to words with English neighbors were faster than to English hermit words. In contrast, the effect of *Dutch Neighbors* was not significant, and neither was the interaction between *English Neighbors* and *Dutch Neighbors* (removed from the final model). Moreover, RTs were faster when a target word’s English frequency was high, but not when the word had a high English bigram frequency in addition to a high English word frequency. Finally, participants responded more slowly when their RT to the previous trial was long.

For non-words, the final regression model in **Table 7** revealed the opposite pattern with respect to neighbor effects: If a word has English neighbors, this slowed down responses, while having Dutch neighbors led to faster responses. Note that although the effect of *Dutch Neighbors* again was not significant, a trend toward facilitation was observed. Finally, similar to what was observed in the word data, non-words with a higher English bigram frequency elicited longer RTs, and non-words were responded to slower when the RT to the previous trial was high.

### Discussion

Comparable to Experiment 1 with the large vs. small neighborhood manipulation, responses to words with English neighbors were faster than to English hermit words in Experiment 2. In contrast, there was no significant main effect or interaction effect of *Dutch Neighbors*. For the non-words in the task, the opposite pattern arose with respect to neighbor effects: If a non-word had English neighbors, this slowed down responses, while having Dutch neighbors led to faster responses. Again, the effect of the number of *Dutch Neighbors* was not significant, but there was a trend toward this behavior. Finally, the word and non-word analysis showed interaction or main effects of English bigram frequency, respectively: A higher English bigram frequency led to slower responses to non-words overall, possibly because it seemed more likely that the item was an English word. A higher English bigram frequency led also to slower responses to English words when the English word frequency was high, which might be due to the additional effect of English bigram frequency being smaller for words with a higher English frequency than for words with a lower English frequency.

Although we did not observe a significant effect of Dutch neighborhood density, the direction of Dutch neighborhood density effects replicates that observed by van Heuven et al. (1998). We note that the null effects of the non-target language Dutch (L1) in the present English (L2) specific circumstances are in line with the often observed null-effects for interlingual homographs in purely ELD experiments. For instance, when only English items and interlingual homographs were incorporated in an ELD study with Dutch–English bilinguals, Dijkstra et al. (1998), (Experiment 1) did not observe any effect of the Dutch reading of the homograph on its ELD time. However, when Dutch words, requiring a ‘no’-response, were added to the stimulus list (in Experiment 2), a strong inhibition effect arose for the interlingual homographs.

Together, the word and non-word results indicate that in our hermit experiment, the target language English (L2) was a more influential language than Dutch (L1). This result was different for the words in van Heuven et al. (1998) and in replication Experiment 1 (see **Table 2**). In those experiments, the English within-language neighborhood size manipulation had an effect of only a few ms. In contrast, it was *Dutch Neighbors* that did most of the work in van Heuven et al. (1998). For non-words, analysis of both studies showed a facilitation effect for non-words without neighbors in English compared to non-words with neighbors in English or in both languages. This indicates that English word neighbors were activated and competed for selection, slowing down rejection of the non-word. However, as can be seen in **Table 3** (cf. **Table 6** in van Heuven et al., 1998), the contribution of English relative to Dutch neighbors to non-word RTs was smaller than in our hermit experiment [the contribution of English and Dutch neighbors in van Heuven et al. (1998) was 16 ms and 9 ms, respectively, vs. in our hermit experiment 44.5 ms and -11.5 ms].

This finding suggests that English plays a much more important role in our ELD experiments with neighbors and hermits than in van Heuven et al. (1998). This is further underlined by our finding that the RT differences between the word and non-word conditions with both English and Dutch neighbors were statistically non-significant from those with only English neighbors (594 ms vs. 599 ms). In contrast, in van Heuven et al. (1998) items with both English and Dutch neighbors were responded to slower than items with only English neighbors (585 ms vs. 561 ms), but their RTs were non-significantly different from those to items that had only Dutch neighbors (585 ms vs. 583 ms).

If our result patterns can be replicated in another task, this will provide support the view that the results are not task-dependent and in line with the general conclusion that the target language English (L2) in our hermit manipulation is stronger relative to Dutch (L1). However, we note that both Dutch (Experiment 1) and English (Experiment 2) neighbors are activated and effective in our participant group of Dutch students, but under different experimental conditions.

## Experiment 3: English Progressive Demasking

For the purposes of cross-task comparison and independent confirmation, we next conducted an EPDM task using the words of Experiment 2. In this task, the target item is gradually demasked and must be reported as quickly as possible by the participant. In van Heuven et al. (1998) effects for English words in blocked PDM (Experiment 1) were quite different in size and pattern from those in ELD (Experiment 4), as can also be seen by comparing our **Tables 2**, **8**. van Heuven et al. (1998) (in their **Table 7**) reported facilitatory English neighborhood size effects in ELD of 3 ms and in blocked EPDM of 34 ms; and inhibitory Dutch neighborhood size effects in ELD of 22 ms and in blocked EPDM of 57 ms (ignoring rounding errors). Thus, neighborhood effects are more visible in progressive demasking due to the nature of the task: The alternation of the mask in this task results in longer RTs than in lexical decision. Using our stronger hermit manipulation, we therefore replicated this experiment as well, also to obtain independent confirmation that effects of English neighborhood are indeed stronger than 20 years ago.

### Method

#### Participants

Twenty-nine Dutch L2 speakers of English (mean age 22.3 years old, *SD* = 2.96), mostly undergraduates at the University of Nijmegen, were paid or received course credits to take part in this experiment. All were highly proficient in English, having learned English from the age of 11 onward. All had normal or corrected-to-normal eyesight.

#### Materials

We used the same 105 English words as in Experiment 1. No non-words were used in this experiment.

#### Procedure

Participants performed an EPDM task, which was programmed in Presentation v13.0 (Neurobehavioral Systems)^4^ and run on an HP Compaq Intel Core 2 computer with LCD monitor. Participants were seated at a table at a 60 cm distance from the computer screen. Stimuli were presented in white capital letters (24 points) in font Arial in the middle of the screen on a black background. Participants were tested individually in a sound-proof room. They first read English instructions that asked them to push the ‘Enter’ button with their right index finger as soon as they had identified the English word that appeared from a mask of hash tags. They were asked to react as accurately and quickly as possible.

Each trial started with the presentation of a black fixation point ‘+’ in the middle of the screen for 700 ms followed by an empty black screen of 300 ms. Then a mask of four or five hash marks (depending on the length of the stimulus) was presented, followed immediately by the stimulus. The presentation of mask and stimulus were alternated in a progressive cycle: On each cycle, the presentation of the stimulus was increased with 14 ms while the presentation of the mask decreased by 14 ms. The total duration of the cycle remained constant at 350 ms. On the first presentation cycle, the mask was presented for 336 ms and the stimulus for 14 ms. There was no time interval between presentation cycles. The alternation of hash marks and stimuli continued until the subject pressed the ‘Enter’ button or until the stimulus had the maximal duration of 350 ms (26 cycles). After pressing the ‘Enter’ button, the stimulus disappeared from the screen, and participants typed in the word they word they thought to have identified, using the keyboard buttons. There was no time pressure in typing the word. A new trial was started when the participants pressed the ‘Enter’ button again.

The experiment was divided in two blocks of about equal length (block 1: 53 trials, block 2: 52 trials). The first block was preceded by 6 practice trials. Next, the participant could ask questions before continuing with the test trials. Each block began with three dummy trials to avoid lack of attention during the beginning of the two blocks. The end of the first block was indicated by a pause screen. The experiment took approximately 8 min.

After the experiment, participants performed the LEXTale-task (Lemhöfer and Broersma, 2012) to obtain a general measure of proficiency in terms of vocabulary knowledge. The mean score on the LEXTale-task was 95% (range 83.3–100%). Based on their scores, all participants were considered as highly proficient.

### Results

Data cleaning was first carried out based on the error rate for participants. Mean participant accuracy on word items was 98.4% (range 95–100%). Next, no items had to be excluded based on their error rate (mean accuracy: 98.4%, range 82.8–100%). A response was classified as an error if a word other than the target word was typed. Obvious cases in which a wrong keyboard button was pressed were not counted as errors. No word items had to be excluded. RTs from incorrect or null responses were removed from the data (2.04% of the 3045 data points) for RT analyses. Outlier RTs above or below 2.5 SD from the item or participant mean (1.9% of the remaining data points) were also removed. This resulted in a data set with 2926 data points.

**FIGURE 4:**
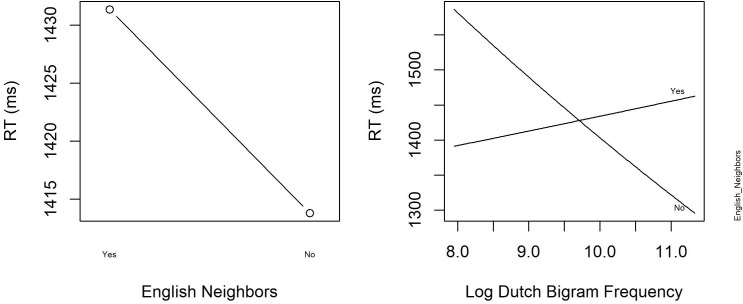
The significant main effect of English Neighbors and the interaction between English Neighbors and Log Dutch Bigram Frequency in English Progressive Demasking with hermits (Experiment 3).

Similar to Experiment 1, RTs were analyzed by means of linear mixed effects models with subject and item as crossed random effects. We considered the same factorial and continuous predictors as in Experiment 1. The logarithmically transformed RT provided the best fit to the data, and therefore, RTs were log-transformed. Finally, the same procedure as in Experiment 1 was applied to obtain the best fitting model. **Table 8** summarizes the mean RTs (in ms) and standard deviations for English word stimuli of this study and of the blocked PDM Experiment 1 of van Heuven et al. (1998). The results for van Heuven et al. (1998) are the average RTs across all their participants (see their **Table 3**). van Heuven et al. (1998) do not indicate overall mean SDs (between 185 and 314) and error rates (between 2.0 and 5.5 %). **Table 9** presents the coefficients of the final model, together with the standard deviation and *t*-value. **Figure 4** displays the main effect of *English Neighbors* and the significant interaction of *English Neighbors* and *Dutch Bigram Frequency*.

**Table 8:** Mean RTs (in ms), standard deviations, error rates, and neighborhood effects for English word stimuli of blocked English Progressive Demasking in van Heuven et al. (1998, Experiment 1) and our Experiment 3 with hermits.

Language effect in van Heuven et al. (1998): English Progressive Demasking	Large English	Small English	Effect size for English	Total effect size for English	
Large Dutch	1666 (205, 2.6)	1652 (209, 3.1)	14	
Small Dutch	1558 (199, 3.9)	1640 (221, 3.2)	-82	-34
Effect size for Dutch	108	12			
Total effect size for Dutch	60			

**Language effect in Experiment 3: English Progressive Demasking**	**Large English**	**No English**	**Effect size for English**	**Total effect size for English**

Large Dutch	1453 (253, 1.5)	1520 (298, 0.7)	-67	
No Dutch	1468 (293, 2.5)	1492 (270, 1.3)	-24	-45.5
Effect size for Dutch	-15	28		
Total effect size for Dutch	6.5			


**Table 9:** Final model for Experiment 3 (English Progressive Demasking with hermits).

Fixed effects	Estimate	Standard error	*t*-value
Intercept	6.242	0.289	21.624
Previous RT	0.075	0.017	4.563
Trial	-0.015	0.008	-2.046
English Frequency	-0.075	0.012	-6.149
English Neighbors	0.724	0.305	2.373
Dutch Bigram Frequency	0.014	0.029	0.511
English Bigram Frequency	0.064	0.023	2.815
English Neighbors by Dutch Bigram Frequency	-0.075	0.031	-2.408

**Random effects**	**Variance**	**Standard deviation**	

Item (Intercept)	0.006	0.076	
Participant (Intercept)	0.032	0.178	
Trial (Participant)	0.001	0.035	
Residual	0.038	0.195	


*t > 1.96 or <-1.96 is significant. Final model: invRT∼ Previous RT + Trial + English Frequency + English Neighbors ^∗^ Dutch Bigram Frequency + English Bigram Frequency + (1| participant) + (1| item). On the intercept are words with no English and Dutch neighbors.*

**Figure 4** reveals that words with English neighbors are responded to faster than words without English neighbors (English neighborhood facilitation). Moreover, RTs to words without English neighbors are slower when their Dutch bigram frequencies are high (i.e., when they resemble Dutch words), whereas for words with many English neighbors, a high Dutch bigram frequency leads to faster responses. Finally, the significant main effects of *English Frequency*, *Trial* and *Previous RT* show, respectively, that participants respond faster when the frequency of a word is high, that they become faster throughout the experiment, and that they respond slower when the RT to the previous word was large.

### Discussion

The RT pattern of the word data in EPDM with hermits shown in **Table 8** largely replicates the pattern of effects in ELD (Experiment 2): RTs to words with neighbors in the target language (English) were faster than to hermit words. The variable *Dutch Neighbors* did not reach statistical significance and was therefore not included in the final model.

Thus, in our experiment, having or not having English neighbors was more important than having or not having Dutch neighbors. This suggests that participants in our study considered English as the default language for executing this task and were not expecting Dutch words. Nevertheless, Dutch activation was shown to affect English word processing. For words that did not have neighbors in English (i.e., English hermits), a high Dutch bigram frequency was not helpful and slowed down word processing. Apparently, the identification of these words could not be facilitated by additional activation of English neighbors and was hindered by the orthographic similarity to Dutch. Thus, the similarity to Dutch added to the insecurity in the processing of identifying an English word. This interaction with Dutch bigram frequency therefore suggests that there is some early lexical activation linked to language information (cf. Midgley et al., 2008). For words with many English neighbors, a high Dutch bigram frequency did not result in a slower identification of the English target word, possibly because the activated English neighbors were already contributing to the identification of the correct target word.

These results partially replicate those obtained by van Heuven et al. (1998) in their blocked Progressive Demasking task (see **Table 8**). Again, the RTs in the condition with many English and many Dutch neighbors were non-significantly faster than those in the condition with only English neighbors (1468 ms vs. 1453 ms). In van Heuven et al. (1998) the many/many condition was just as fast the condition with only Dutch neighbors, at least for their High Proficiency participants (1669 ms vs. 1670 ms). A large number of neighbors in both languages slowed down the RTs for their Low Proficiency participants.

A comparison of the effects in the two studies again shows that English in our study had stronger effect than Dutch. Facilitation effects for English increased relative to van Heuven et al. (1998) whereas inhibition effects for Dutch decreased and even became non-significant.

We further note that the RTs in our EPDM experiment were considerably faster than those in that by van Heuven et al. (1998) even relative to their high proficiency bilinguals (see **Table 8**). At the same time, like in lexical decision, English neighborhood size had a facilitatory effect in both studies, whereas Dutch neighborhood size had more of an inhibitory effect in both studies, although in our study this was limited to complete hermits vs. words with only Dutch neighbors.

On the whole, the RT patterns we obtained in progressive demasking and lexical decision appear to be rather similar. This suggests that the observed result patterns are probably due to more central aspects of lexical activation rather than to a decision stage, which is in line with the suggestions by Midgley et al. (2008) based on EEG data that there is a pre-decision component to neighborhood effects. In the General Discussion, we will interpret these results by arguing that target word retrieval is sensitive to language-specific global lexical activation and lexical competition between word candidates. Like in ELD, in EPDM, global activation of neighbors in the target language apparently again facilitates target word retrieval, while that in the non-target language produces interference.

## General Discussion

As a first aim of the present study, we investigated the hypothesis that neighborhood effects are sensitive to L2 proficiency, because it changes the relative prominence of the native and the second language. Experiment 1 replicated the ELD experiment on cross-linguistic neighborhood effects in a well-known earlier study (van Heuven et al., 1998, Experiment 4). In line with our hypothesis, our bilingual participants, who can be considered as the next generation of the earlier population contributing to van Heuven et al. (1998) performed the ELD task 40–60 ms faster. Furthermore, in contrast to the earlier study, in which effects of Dutch dominated the result patterns, the new experiment led to a predominance of English. We observed a different result pattern than in the earlier study, with prominent effects of English neighbors and no effects of Dutch neighbors. We argued that this a consequence of the increased proficiency level in English of our participants compared to that of the participants of 20 years earlier.

As a second aim of our study, we confirmed and extended these results by conducting a new ELD experiment with a stronger neighborhood manipulation. Experiment 2 compared the performance on English target words and non-words without any neighbors in English (L2) and Dutch (L1) to that of words and non-words that either had neighbors in English or Dutch, or in both languages. Such items without any neighbors in one or two languages, called *hermits*, provide a much more contrastive manipulation than items with many or few neighbors. We found that English words with only Dutch neighbors were responded to slower and less accurately than English words that were complete hermits. At the same time, non-words with only Dutch neighbors were rejected faster than complete hermit non-words. Thus, in our ELD task with the current participant population, English, including English neighbors, dominated the RT patterns.

As a third aim of our study, we tested the stability of obtained result patterns across tasks, given that the results of earlier studies (e.g., van Heuven et al., 1998; Dirix et al., 2016) varied considerably across experimental paradigms. In Experiment 3, we included the stimulus words in EPDM with other participants of the same population. The neighborhood manipulation in terms of hermit characteristics led to comparable result patterns across the two hermit experiments of our study and can be summarized in terms of two observations (for reference, see **Tables 2**, **8**).

First, in both our tasks, English words with only English neighbors were responded to faster and more accurately than English hermits in both languages. Non-words with only English neighbors, however, were rejected much slower than complete hermit non-words. The same pattern of results was found by van Heuven et al. (1998) in their progressive demasking task, but in their lexical decision study, there was only an unexpected non-significant 3 ms difference for the words in the two conditions.

In our progressive demasking task, there also was no significant effect of Dutch neighborhood density, but a large Dutch bigram frequency slowed down identification of the English target word. Such effects of Dutch on English are in line with the stronger inhibitory influences of Dutch on English words in the ELD and progressive demasking experiments by van Heuven et al. (1998). Thus, in our participant population, we obtain effects of both Dutch and English neighbors, but the effects are dependent on stimulus list composition and task demands.

The second observation is concerned with the responses to words with many neighbors in both languages and words with neighbors in only one language. In both tasks, we observed that the word responses in this condition were *non-significantly different in speed and accuracy* from those in the condition with only English neighbors. This also holds for the non-words in the lexical decision task. This finding also confirms that for both English tasks and participant groups in our study, English was more prominent than Dutch. Here lies a clear difference relative to the study by van Heuven et al. (1998) where the responses in the condition with many neighbors in both languages mostly aligned with those in the condition with only Dutch neighbors.

In sum, we conclude that the comparable prominence of English in our three experiments, involving neighbors and hermits, can be ascribed to the high L2 proficiency of the current generation of Dutch-English bilinguals. However, as another factor that may have contributed to the findings of the hermit experiments, we note the following difference in experimental manipulation: Dutch neighbors of the English target words were absent in two of the four hermit conditions (the ‘no Dutch’-conditions), but present in all of the standard neighbor conditions (both the ‘large Dutch’ and the ‘small Dutch’ conditions).

As the fourth and final aim of our study we wished to clarify both theoretical and empirical issues with respect to neighborhood studies. From a theoretical perspective, the comparison of the two neighborhood and task types in the present study, as well as their comparison to van Heuven et al. (1998) allowed us to analyze the processing mechanisms underlying performance in more detail. We reasoned that an analysis of these might also be helpful to account for some of the fragile neighborhood effects that van Heuven et al. (1998) and Dirix et al. (2016) obtained in their empirical studies. We will discuss these two points in turn.

Two mechanisms that are often proposed to play a role in visual word processing are lexical competition and global lexical activation. Interactive activation models for bilingual word recognition, such as BIA and BIA+ (Dijkstra and van Heuven, 2002), assume inhibitory links between words in the lexicon, resulting in lexical competition effects. When an input word is presented, word candidates in the lexicon are activated depending on word frequency and orthographic similarity to the input. Activated word candidates compete for selection and decrease the activation level of other words, irrespective of the language they belong to, via lateral inhibition. According to such models, activation of both target and non-target language neighbors should inhibit target language word processing. Our three experiments did not show strong inhibition effects for between-language neighbors, but mainly effects of within-language neighbors. Our finding that within-language neighborhood size facilitates word processing contradicts the models’ prediction and cannot be straightforwardly accounted for. This was already pointed out by van Heuven et al. (1998).

Grainger and Jacobs (1996) argued that apart from lexical competition, a word response can be based on global lexical activation. Their multiple read-out model (an extension of the IA-model) was able to simulate the facilitatory effects of within-language neighborhood size when lexical decisions were based on a response criterion set on summed lexical activity. When more neighbors are activated, there is more global lexical activation, resulting in faster RTs. However, we did not obtain a significant RT difference between words with only English neighbors and neighbors in both languages. If it were the summed activation of both activated English and Dutch neighbors that triggered a response, then mean RTs to words with both English and Dutch neighbors should be significantly slower than to words with only English neighbors. The activated Dutch neighbors would then reduce the facilitatory effects caused by the activation of the English neighbors.

Our hermit results can be explained by a combination of lexical competition and global activation, if it is assumed that lexical decisions or lexical retrieval times in progressive demasking are affected most by the summed activation of word candidates in the language relevant for the task at hand. In other words, summed lexical activation should be based on summed language-specific lexical activation rather than on activation across languages, at least when the task is language-specific lexical decision or progressive demasking. Within the framework of the BIA+ model, such language membership information is collected by language nodes that add up the activation of active lexical possibilities. In the model, these language-specific sets of activated lexical items can then affect response selection. For example, in an ELD task with Dutch–English bilinguals, both the activated English target word and its activated Dutch and English neighbors will activate their language membership information at the language node. Therefore, for the hermit manipulation, Dutch will be activated to a smaller extent than for the standard neighbor manipulation. According to BIA+, the activated language nodes will feed the response mechanism in the task-decision system in order to build up the probability for a given response. English word candidates will collectively activate the yes-response, whereas Dutch word candidates will activate the no-response. When the appropriate word has been selected (lexical competition is resolved) or a large amount of global activation has reached a set threshold, the task-decision system will weigh this input, which is linked to a certain response, against the activation that has already been built up in favor of the other response, and the response is selected.

Because task differences between progressive demasking and lexical decision did not affect the major result patterns in our study, this supports the notion from Midgley et al. (2008) that it is the selection of the right word that is affected by both lexical competition and language-specific global lexical activation, rather than the response (yes or no) itself.

The interpretation of our results in terms of the mechanisms of lexical competition and global activation is relatively straightforward, and it seems likely that the same mechanisms also play a role in other studies, like those by van Heuven et al. (1998) and Dirix et al. (2016). In fact, the considerable differences in results reported across studies (see Ferrand, 2001, pp. 97–98, for a review) indicate that neighborhood effects are quite sensitive to details of experimental design, stimulus characteristics, and stimulus list context (as also argued by Van Kesteren et al., 2012).

## Conclusion

In three experiments, we considered four issues in the domain of within-language and between-language orthographic neighborhood effects. First, we predicted a shift in the predominance of languages (from Dutch to English) for a replication of a cross-linguistic neighbor study conducted 20 years ago, based on the observation that the English proficiency of the participants has meanwhile increased considerably. The obtained result patterns were in line with this prediction. Second, we conducted a stronger manipulation of neighborhood density by resorting to a hermit contrast. This manipulation confirmed the earlier effects of English neighborhood density in the participant population. In all, the contrast between complete hermit words and non-words with items having neighbors in only one language or both the target- and non-target language provided a “cleaner” and stronger measurement of neighborhood size effects.

Third, given that the results of earlier studies (e.g., van Heuven et al., 1998; Dirix et al., 2016) varied considerably across experimental paradigms, we tested the stability of obtained result patterns across different tasks with exactly the same materials and design. We obtained convergent result patterns for two unilingual tasks also used by van Heuven et al. (1998) language-specific ELD and progressive demasking.

Finally, we analyzed our study in terms of the processing mechanisms underlying neighborhood effects in the two tasks. We found that the selection of a target word is affected by both lexical competition and language-specific global lexical activation. Models of bilingual word recognition, like BIA+, should include both of these general mechanisms. Because global lexical activation per language is captured in the BIA+model by means of language nodes, the importance of these as a factor contributing to the result patterns must be stressed.

## Ethics Statement

This study was carried out in accordance with the recommendations of Ethics Committee Social Sciences of Radboud University Nijmegen (permission is granted to TD under number ECG2012-2711-05a, Language Processing in Multilinguals) with written informed consent from all subjects. All subjects gave written informed consent in accordance with the Declaration of Helsinki. The protocol was approved by the Ethics Committee Social Sciences.

## Author Contributions

KM: data collection, data analyses and interpretation, and wrote the paper. WvH: help with data analysis and interpretation of the data. TD: data interpretation and wrote the paper.

## Conflict of Interest Statement

The authors declare that the research was conducted in the absence of any commercial or financial relationships that could be construed as a potential conflict of interest.

## Footnotes

1 We reanalyzed the data originally reported by van Heuven et al. (1998) with linear mixed effects models to assess the robustness of the effects that were reported in their ANOVAs. As in the original analyses, only English Neighbors (Large/Small) and Dutch Neighbors (Large/Small) were included as predictors. Analyses of the word data revealed a significant inhibitory effect of Dutch neighborhood size on response latencies (*t* = 2.407, *p* = 0.0187) and no effect of or interaction with English neighborhood size. The analyses of the non-word data showed only a trend towards an inhibitory effect of English neighborhood size (*t* = 1.50, *p* = 0.138).

2 An analysis of the word and non-word data of Experiment 1 and the original data by van Heuven et al. (1998) combined, including the predictors Generation (1998/2018), Dutch Neighbors (Large/Small), and English Neighbors (Large/Small) showed significant interactions between Generation and Dutch Neighbors in both the word and non-word data. The interactions show that the participants of van Heuven et al. (1998) responded more slowly than our participants to English words and non-words when Dutch neighborhood size was large. In other words, Dutch was much more prominent for the earlier participants.

3 www.nbs.com

4 www.nbs.com

## APPENDIX

**Stimulus materials of all experiments**.

**Experiment 1: English lexical decision with neighbors**

Words in Experiment 1

*Small N English/Small N Dutch:* deny, duty, earl, envy, evil, folk, frog, guts, idol, kiss, okay, oral, oval, soup, true, twin, ugly, used, vein, view
*Small N English/Large N Dutch:* army, atom, bias, bird, diet, edge, germ, huge, butt, jerk, keen, knee, liar, lion, myth, noon, nude, obey, poem, poor
*Large N English/Small N Dutch:* bath, bomb, busy, clue, coin, desk, dial, dirt, dish, firm, gray, hurt, iron, joke, lamb, limb, loss, milk, prey, rude *Large N English/Large N Dutch:* aunt, blue, farm, hawk, knit, left, loan, loud, maid, monk, moon, path, quit, shoe, suit, tool, verb, weak, wrap, zero

Non-words in Experiment 1

*Small N English/Small N Dutch:* drio, frig, jofe, kach, kiot, knaf, luet, maup, moug, nige, omil, paby, ridi, siom, taur, torp, tuni, unar, zous, zuke
*Small N English/Large N Dutch:* bito, grul, jees, jeul, kalp, keun, morp, mups, nazz, nont, noto, oune, pris, puif, reug, reun, slen, viem, woup, zuls
*Large N English/Small N Dutch:* jant, lurp, lusp, naul, nirk, nudo, orim, pani, prad, prog, puet, raut, reud, rion, seto, snam, tirk, tran, vich, vorn
*Large N English/Large N Dutch:* aunk, blag, boul, boup, bret, dris, duef, elap, fram, frip, furk, gonk, jeef, knat, knub, koup, loem, mots, rama, sluk
**Experiment 2 (English lexical decision with hermits) and Experiment 3 (English progressive demasking with hermits)**

Words in Experiment 2 and Experiment 3

When applicable, the number of orthographic neighbors of an item in English and/or Dutch is indicated between parentheses.
*Complete hermits:* arrow, sugar, digit, exist, raise, equal, doubt, faith, empty, ghost, attic, first, abbey, elbow, knife, often, posit, eagle, habit, ivory, asset, evoke, lunar, proud, razor, maybe, merit, amaze, envy, glory
*Words with only Dutch neighbors:* movie (1), guard (1), fluid (2), hazel (4), cabin (1), proof (2), clerk (3), lapse (1), erupt (1), urge (11), obey (1), view (3), liar (4), void (1), bias (11)
*Words with only English neighbors:* queen (3), ample (3), nurse (3), cheap (2), debt (2), apply (3), angle (1), couch (6), share (15), cheek (4), smoke (3), snake (5), float (3), mouth (4), scarf (5), judge (3), peach (7), drown (5), porch (6), chest (3), skill (5), haunt (5), brush (3), allow (3), gorge (4), goose (4), nasty (4), cloud (2), faint (5), noise (4)
*Words with English and Dutch neighbors:* duck (15/6), wood (10/8), hawk (4/3), quit (5/12), bird (4/3), dish (5/1), grow (6/6), tune (7/4), blame (5/1), spoon (5/3), shift (3/3), pride (6/4), glove (3/2), sheet (6/4), paint (6/3), scare (9/2), space (5/1), brake (6/1), candy (7/2), swamp (3/2), plain (2/2), eager (3/6), burst (2/4), grace (8/3), fever (5/7), spoil (1/1), alley (2/2), light (9/1), layer (3/5), power (10/3)

Non-words used in Experiment 2 (English lexical decision with hermits).

When applicable, the number of orthographic neighbors of an item in English and/or Dutch is indicated between parentheses.
*Complete hermit non-words:* gaish, leith, imary, ghorf, rasle, pafle, redle, emare, asame, roilt, muzor, umpsy, swufe, faige, orult, sopit, huser, teyal, jeish, cloif, fleap, lerme, prerg, moash, ecose, halic, doolp, exape, togar, irfe
*Non-words with only Dutch neighbors:* zorf (3), yoos (10), pois (7), etin (2), klig (6), volge (2), frets (5), heish (1), nerst (5), darst (3), sholp (1), hegen (16), lugen (6), garst (4), spuld (1), emmet (1), gleip (1), lilve (1), noret (2), smoog (2), gluit (2), slewe (1), ploag (2), vagel (5), vraig (1), vorg (10), gronc (1), vroog (4), slaip (2), knoog (2)
*Non-words with only English neighbors:* fosh (9), sish (7), aboke (2), lidge (5), mourt (3), traim (3), roash (2), monch (4), creal (3), redge (5), blusp (1), cheem (3), guare (2), touse (6), cloul (2), launt (6), flage (4), vitch (7), daint (5), tenal (4), hount (5), goast (4), noght (1), dable (5), wheeg (1), sooth (5), douth (5), waish (2), parsh (4), swame (3)
*Non-words with English and Dutch neighbors:* dosk (6/6), hade (12/9), pilt (13/10), mair (8/6), greel (6/5), dorse (8/4), pleat (3/3), lawer (4/5), haron (2/3), gleep (2/2), groap (3/1), scole (6/2), scade (5/2), prood (3/2), dight (11/1), bover (9/10), metel (4/9), claip (3/1), tonus (2/2), swion (2/1), lavel (6/9), avone (2/1), smoop (5/5), prail (3/1), blear (3/1), vaber (2/2), twiss (2/1), malve (5/2), spoit (5/4), rasel (2/4)

**Table A1:** Characteristics of the word and non-word items in Experiments 2 and 3 (data entered into the analysis).

	Stimulus category	Length	Log Frequency	Log English bigram	Log Dutch bigram	English neighbors	Dutch neighbors
Word	Complete hermit (29)	4.97	2.81	8.73	9.80	0	0
	Only Dutch neighbors (14)	4.57	2.72	8.43	9.94	0	2.57
	Only English neighbors (29)	4.97	2.95	8.79	9.88	4.24	0
	Neighbors in English and Dutch (30)	4.73	3.06	8.76	9.87	5.07	3.4
Non-word	Complete hermit (30)	4.97	–	8.73	9.76	0	0
	Only Dutch neighbors (30)	4.83	–	8.67	10.03	0	3.63
	Only English neighbors (27)	4.93	–	8.84	9.87	3.85	0
	Neighbors in English and Dutch (28)	4.86	–	8.88	10.02	4.79	3.66	

## References

[B1] Baayen R. H. Piepenbrock R. Gulikers L. (1995). *The CELEX Lexical Database [CD-ROM].* Philadelphia, PA: University of Pennsylvania Linguistic Data Consortium.

[B2] Balota D. A. Yap M. J. Cortese M. J. Hutchison K. A. Kessler B. Loftis B. (2007). The english lexicon project. *Behav. Res. Methods* 39 445–459. 10.3758/BF03193014 17958156

[B3] Bowers J. S. Davis C. J. Hanley D. A. (2005). Interfering neighbors: the impact of novel word learning on the identification of visually similar words. *Cognition* 97 45–54. 10.1016/j.cognition.2005.02.002 15925358

[B4] Brysbaert M. New B. (2009). Moving beyond Kučera and Francis: a critical evaluation of current word frequency norms and the introduction of a new and improved word frequency measure for American English. *Behav. Res. Methods* 41 977–990. 10.3758/BRM.41.4.977 19897807

[B5] Coltheart M. Davelaar E. Jonasson J. T. Besner D. (1977). “Access to the internal lexicon,” in *Attention and Performance*, ed. Dornic S. (London: Academic Press), 535–555.

[B6] Davis C. J. Andrews S. (1996). “The role of computational modeling in studies of visual word recognition,” *Symposium on Computer Models of Cognition: Possibilities and Pitfalls, 31st Annual Conference of the Australian Psychological Society*, Sydney, September 25–29.

[B7] De Swaan A. (2001). *Words of the World. The Global Language System.* Cambridge: Polity Press.

[B8] Dijkstra T. (2007). “The multilingual lexicon,” in *Handbook of Psycholinguistics*, ed. Gaskell G. (Oxford: Oxford University Press), 251–265.

[B9] Dijkstra T. Miwa K. Brummelhuis B. Sappelli M. Baayen H. (2010). How cross-language similarity and task demands affect cognate recognition. *J. Mem. Lang.* 62 284–301. 10.1016/j.jml.2009.12.003

[B10] Dijkstra T. van Heuven W. J. B. (2002). The architecture of the bilingual word recognition system: from identification to decision. *Biling. Lang. Cogn.* 5 175–197. 10.1017/S1366728902003012 15905078

[B11] Dijkstra A. T. Van Jaarsveld H. Ten Brinke S. (1998). Interlingual homograph recognition: effects of task demands and language intermixing. *Biling. Lang. Cogn.* 1 51–66. 10.1017/S1366728998000121

[B12] Dirix N. Cop U. Drieghe D. Duyck W. (2016). Cross-lingual neighborhood effects in generalized lexical decision and natural reading. *J. Exp. Psychol. Learn. Mem. Cogn.* 43 887–915. 10.1037/xlm0000352 28095009

[B13] Duyck W. Desmet T. Verbeke L. Brysbaert M. (2004). WordGen: a tool for word selection and nonword generation in Dutch, German, English, and French. *Behav. Res. Methods Instrum. Comput.* 36 488–499. 10.3758/BF03195595 15641437

[B14] Edelenbos P. Vinjé M. P. (2000). The assessment of a foreign language at the end of primary (elementary) education. *Lang. Test.* 17 144–162. 10.1177/026553220001700203

[B15] Ferrand L. (2001). *Cognition et Lecture: Processus de Base de la Reconnaissance Des Mots Écrits Chez l ’Adulte.* Brussels: DeBoeck & Larcier.

[B16] Grainger J. Jacobs A. M. (1996). Orthographic processing in visual word recognition: a multiple read-out model. *Psychol. Rev.* 103 518–565. 10.1037/0033-295X.103.3.518 8759046

[B17] Grossi G. Savill N. Thomas E. Thierry G. (2012). Electrophysiological cross-language neighborhood density effects in late and early English-Welsh bilinguals. *Front. Psychol.* 3:408. 10.3389/fpsyg.2012.00408 23087661 PMC3475346

[B18] Holcomb P. J. Grainger J. (2007). Exploring the temporal dynamics of visual word recognition in the masked repetition priming paradigm using event-related potentials. *Brain Res.* 1180 39–58. 10.1016/j.brainres.2007.06.110 17950262 PMC2151932

[B19] Holcomb P. J. Grainger J. O’Rourke T. (2002). An electrophysiological study of the effects of orthographic neighborhood size on printed word perception. *J. Cogn. Neurosci.* 14 938–950. 10.1162/089892902760191153 12191460

[B20] Keuleers E. Brysbaert M. (2012). “Detecting inherent bias in lexical decision experiments with the LD1NN algorithm,” in *Methodological and Analytic Frontiers in Lexical Research*, eds Libben G. Jarema G. Westbury C. (Amsterdam: John Benjamins Publishing), 231–248.

[B21] Kounios J. Holcomb P. J. (1992). Structure and process in semantic memory: evidence from event-related brain potentials and reaction times. *J. Exp. Psychol. Gen.* 121 459–479. 10.1037/0096-3445.121.4.459 1431739

[B22] Kutas M. Federmeier K. D. (2000). Electrophysiology reveals semantic memory use in language comprehension. *Trends Cogn. Sci.* 4 463–470. 10.1016/S1364-6613(00)01560-6 11115760

[B23] Kutas M. Hillyard S. A. (1980). Reading senseless sentences: brain potentials reflect semantic incongruity. *Science* 207 203–205. 10.1126/science.7350657 7350657

[B24] Lemhöfer K. Broersma M. (2012). Introducing LexTALE: a quick and valid lexical test for advanced learners of English. *Behav. Res. Methods* 44 325–343. 10.3758/s13428-011-0146-0 21898159 PMC3356522

[B25] Meara P. Milton J. (2003). *X_Lex, the Swansea Levels Test.* Newbury: Express.

[B26] Midgley K. J. Holcomb P. J. van Heuven W. J. B. Grainger J. (2008). An electrophysiological investigation of cross-language effects of orthographic neighborhood. *Brain Res.* 1246 123–135. 10.1016/j.brainres.2008.09.078 18948089 PMC2656968

[B27] Mulder K. Schreuder R. Dijkstra T. (2013). Morphological family size effects in L1 and L2 processing: an electrophysiological study. *Lang. Cogn. Process.* 28 1004–1035. 10.1080/01690965.2012.733013

[B28] Oganian Y. Conrad M. Aryani A. Heekeren H. R. Spalek K. (2016). Interplay of bigram frequency and orthographic neighbourhood statistics in language membership decision. *Biling. Lang. Cogn.* 19 578–596. 10.1017/S1366728915000292

[B29] Oganian Y. Conrad M. Aryani A. Spalek K. Heekeren H. R. (2015). Activation patterns throughout the word processing network of L1-dominant bilinguals reflect language similarity and language decisions. *J. Cogn. Neurosci.* 27 2197–2214. 10.1162/jocn_a_00853 26226076

[B30] Ratcliff R. Gomez P. McKoon G. (2004). A diffusion model account of the lexical decision task. *Psychol. Rev.* 111 159–182. 10.1037/0033-295X.111.1.159 14756592 PMC1403837

[B31] Special Eurobarometer 147 (2001). *Europeans and Their Languages. INRA Europe.* Available at: http://ec.europa.eu/commfrontoffice/publicopinion/archives/ebs/ebs_147_en.pdf

[B32] Special Eurobarometer 386 (2012). *Europeans and Their Languages. European Commission: Wave EB77.1 Annex.* Available at: http://ec.europa.eu/commfrontoffice/publicopinion/archives/ebs/ebs_386_anx_en.pdf

[B33] Van Hell A. G. (1998). *Cross-Language Processing and Bilingual Memory Organization.* Doctoral dissertation, University of Amsterdam, Amsterdam.

[B34] van Heuven W. J. Dijkstra T. Grainger J. (1998). Orthographic neighborhood effects in bilingual word recognition. *J. Mem. Lang.* 39 458–483. 10.1006/jmla.1998.2584

[B35] Van Kesteren R. Dijkstra T. De Smedt K. (2012). Markedness effects in Norwegian-English bilinguals: task-dependent use of language-specific letters and bigrams. *Q. J. Exp. Psychol.* 65 2129–2154. 10.1080/17470218.2012.679946 22554207

[B36] Yarkoni T. Balota D. Yap M. (2008). Moving beyond Coltheart’s N: a new measure of orthographic similarity. *Psychon. Bull. Rev.* 15 971–979. 10.3758/PBR.15.5.971 18926991

